# Rasburicase-Induced Falsely Low Measurement of Uric Acid in Tumor Lysis Syndrome: A Report of Two Cases

**DOI:** 10.7759/cureus.34435

**Published:** 2023-01-31

**Authors:** Lian Yu, Yinjing Xie, Jiangtao Ma, Guoqiang Li, Xiaopeng Yuan

**Affiliations:** 1 Department of Medical Laboratory, Shenzhen People’s Hospital, Shenzhen, CHN

**Keywords:** case report, hyperuricemia, tumor lysis syndrome, uric acid, rasburicase

## Abstract

Rasburicase, a recombinant urate-oxidase enzyme, can significantly catalyze the oxidation of uric acid to allantoin. It was approved by the US Food and Drug Administration (FDA) to control blood uric acid levels in both pediatric and adult patients especially those with tumor lysis syndrome. It is quite important to realize that rasburicase can continue to be effective ex vivo and cause falsely low results if the blood sample is not contained and transported in ice water immediately. We presented two cases of falsely low measurement of blood uric acid caused by rasburicase and elaborated the proper method for collecting and transporting blood samples from patients using rasburicase.

## Introduction

Tumor lysis syndrome (TLS) is a life-threatening oncologic emergency that occurs more frequently in patients with hematological malignancies, especially those sensitive to chemotherapy [1]. The initial cytoreductive therapy used for the treatment of the malignancy causing extensive death and lysis of tumor cells may release massive amounts of potassium, phosphate, and nucleic acids into the systemic circulation, resulting in hyperkalemia, hyperphosphatemia, hyperuricemia, and hypocalcemia. These metabolic derangements in TLS may lead to cardiac arrhythmia, acute renal failure, seizures, or even sudden death [2,3].

Hyperuricemia has been reported to be associated with a high risk of TLS-associated acute kidney injury (AKI) [4], which is an independent predictor of mortality in TLS [5]. Elevated serum uric acid can cause kidney damage in several ways, including crystal deposition in tubules, activation of inflammation, renal vasoconstriction and oxidative stress [6]. Thus, reducing uric acid levels effectively is extremely important for improving outcomes in patients with TLS. Rasburicase has a significant effect on the treatment and prevention of hyperuricemia. It can reduce uric acid concentration rapidly and was approved for managing hyperuricemia in TLS by the United States Food and Drug Administration (FDA) in 2002. However, insufficient attention has been paid to the fact that rasburicase can continue to convert uric acid to allantoin ex vivo, and may cause falsely low measurements of uric acid. This situation may have the potential risk of misleading the continued treatment of the patient. The aim of this study was to highlight that rasburicase continues to work ex vivo, so it is important to know the correct method of collecting and transporting specimens to test blood uric acid from patients using rasburicase.

## Case presentation

The first case was a 73-year-old man with a medical history of bacterial pneumonia, chronic kidney disease (CKD) 2, hypertension, depression, prostatic hyperplasia, bladder stones, and subtotal gastrectomy. He was admitted to our hospital for the evaluation of systemic lymphadenopathy, fever, chest tightness, shortness of breath, fatigue, poor appetite, oliguria, and diffuse erythema.

On hospital day 1, he was diagnosed with T-cell lymphoblastic lymphoma by bone marrow biopsy with a large tumor burden and was then treated with chemotherapy. Subsequent blood assessment showed a creatinine value of 548 umol/L (normal: 44-133 umol/L), phosphorus level of 2.36 mmol/L (normal: 0.8-1.5 mmol/L), calcium level of 1.52 mmol/L (normal: 2.05-2.55 mmol/L), and a uric acid level of 1008 umol/L (normal: 90-420 umol/L).

On hospital day 2, our clinical laboratory technicians noticed that his uric acid level decreased from 1008 umol/L to 320 umol/L within 24 hours, so it was retested. However, the results of four tests from the same sample showed decreasing uric acid levels, even approaching zero, as shown in Table 1.

**Table 1:** Uric acid test results of the same serum sample at different time points

Test time	Uric acid (μmol/L)
09:28 am	320
10:14 am	267
12:30 am	115
15:32 pm	1

We checked every work step to ensure nothing was wrong and communicated with the clinician regarding the diagnosis and treatment of the patient. It was noted that due to his diagnosis of lymphoma and large tumor burden, along with a background chronic kidney disease (CKD), hyperuricemia, hyperphosphatemia and hypocalcemia, he was diagnosed as TLS and started on a single dose of rasburicase (4.5 mg) with intravenous fluid (glucose injection) at 17:13 pm on hospital day 1. This indicates that the time interval between rasburicase use and lower uric acid measurement was only approximately 17 hours. The half-life of rasburicase is 15.7-22.5 hours, and it is still active ex vivo. There was a reasonable prospect that rasburicase caused ex vivo degradation and even a rapid drop to almost zero levels of uric acid in the serum sample. Thus, following the second dose of rasburicase, we suggested that the sample needed to be transported in an ice-water bath to avoid false low measurement according to the precautions in drug instructions, and the result of uric acid was 157 umol/L (normal: 90-420 umol/L), while the uric acid level of the sample not transported in ice was zero.

The second case was a 75-year-old man with hypertension and hepatitis B, and had a partial gastrectomy in our hospital due to gastric cancer. He presented with a history of lymph node enlargement for four months and edema in both lower limbs for 10 days. Biopsy suggested "diffuse large B-cell lymphoma". Blood assessment showed a creatinine value of 412 umol/L (normal: 44-133 umol/L), phosphorus level of 1.69 mmol/L (normal: 0.8-1.5 mmol/L), calcium level of 1.87 mmol/L (normal: 2.05-2.55 mmol/L), and a uric acid level of 519 umol/L (normal: 90-420 umol/L). The results of uric acid on different days are shown in Table 2. We checked the patient’s information with his clinician and it turned out that a single dose of rasburicase (4.5 mg) with intravenous fluid (glucose injection) was used at 11.52 pm on March 15, 2022. However, when the sample was recollected immediately and stored in ice, the result of uric acid was 69 umol/L.

**Table 2:** Uric acid test results of different date

Date	Uric acid (μmol/L)
2022.3.14	625
2022.3.15	563
2022.3.16	0
2022.3.16 (recollected sample)	69

## Discussion

Rasburicase is effective in reducing blood uric acid levels. However, clinicians may not pay sufficient attention to the fact that this drug can continue to break down uric acid ex vivo [7]. Thus, for patients using rasburicase to treat hyperuricemia or elevated uric acid levels, blood samples need to be handled correctly.

Rasburicase is a recombinant human uric acid enzyme and temperature has a great influence on its reaction rate. At low temperature, the enzyme activity will decrease. Therefore, to get more accurate uric acid level after using rasburicase, blood samples should be collected and transported at low temperature. First, plasma is collected to measure uric acid levels. Second, the collected blood sample should be contained in a pre-cooled tube with heparin anticoagulant, and then immediately stored in ice water. Finally, the sample is centrifuged in a pre-cooled (4°C) centrifuge to prepare the plasma sample. Fourth, the plasma sample is transported in an ice-water bath. Finally, it is critical that the measurement of plasma uric acid should be conducted within 4 h after blood sample collection.

The risk of death from TLS can increase from 20% to 50% of cases if it is not diagnosed in time [8]. It is potentially fatal and is an emergency that needs close attention and timely treatment. In some cases, the incidence of TLS may cause the patient’s body too bad to withstand chemotherapy, which could save the lives of patients. Therefore, TLS should be managed safely and effectively. A hallmark of TLS is hyperuricemia, which can result in acute kidney injury because uric acid crystals may precipitate in the renal tubule, causing compromised glomerular filtration and obstructive uropathy [9,10]. Since its approval in 2002, rasburicase has been used in a new era for the treatment of hyperuricemia associated with TLS [7]. Currently, it is the standard treatment for patients with TLS [11]. Rasburicase can reduce accumulated uric acid by breaking it down to easily excreted allantoin at a very fast speed [12], and sometimes the blood uric acid levels can even decrease to zero. However, it is critical to note that rasburicase can be active ex vivo. Therefore, clinicians should pay enough attention to choosing the appropriate method to collect and transport samples from patients to avoid rasburicase-caused falsely low measurement of uric acid. We suggest that clinicians should be mentioning whether rasburicase has been used or not when ordering uric acid levels test. It is better that the information system can automatically prompt the sample collection and transportation requirements in this case.

## Conclusions

Rasburicase is a recombinant urate-oxidase enzyme that can effectively reduce blood uric acid levels in TLS patients. However, it continues to be active ex vivo. This report will help inform clinicians of the appropriate method of collecting and transporting samples from patients using rasburicase.
